# The relationship between active travel to school and health-related fitness in children and adolescents: a systematic review

**DOI:** 10.1186/1479-5868-8-5

**Published:** 2011-01-26

**Authors:** David R Lubans, Colin A Boreham, Paul Kelly, Charlie E Foster

**Affiliations:** 1 School of Education, University of Newcastle, Callaghan Campus, Australia; 2 Department of Public Health, University of Oxford, UK; 3 Institute for Sport and Health, University College Dublin, Ireland

## Abstract

**Background:**

Active travel to school (ATS) has been identified as an important source of physical activity for youth. However, the relationship between ATS and health-related fitness (HRF) among youth remains unclear.

**Methods:**

A systematic search of seven electronic databases (EMBASE, OVID MEDLINE, PsycINFO, PubMed, Scopus, SPORTDiscus and TRIS on line) was conducted in December 2009 and studies published since 1980 were considered for inclusion.

**Results:**

Twenty seven articles were identified that explored the relationship between ATS and the following aspects of HRF: weight status/body composition, cardiorespiratory fitness, muscular fitness and flexibility. Forty-eight percent of the studies that examined the relationship between ATS and weight status/body composition reported significant associations, this increased to 55% once poor quality studies were removed. Furthermore, the findings from five studies, including one longitudinal study, indicate that ATS is positively associated with cardiorespiratory fitness in youth. However, the evidence for the relationships between ATS and muscular fitness or flexibility is equivocal and limited by low study numbers.

**Conclusions:**

There is some evidence to suggest that ATS is associated with a healthier body composition and level of cardiorespiratory fitness among youth. Strategies to increase ATS are warranted and should be included in whole-of-school approaches to the promotion of physical activity.

## Background

Higher levels of physical activity are associated with superior physical, social and psychological health in young people. Physical activity is inversely related to overweight and obesity in youth [1,2] and poor body composition in childhood is associated with an increased risk of coronary heart disease in adulthood [3]. While there has been debate about whether or not physical activity levels have declined in the last 30 years [4], evidence suggests that there has been a decline in active travel to school (ATS) among children and adolescents in many countries [5-7]. For example, data from the US National Personal Transportation Survey revealed that the number of students who walked or cycled to school decreased from 41% in 1969 to only 13% in 2001 [6].

ATS includes various modes of travel such as walking, cycling, and skateboarding, and has been identified as an important source of physical activity for young people [8]. Previous reviews [9-11] have examined the relationship between ATS, body composition and physical activity and reported strong evidence for a positive association between ATS and overall physical activity level, but little evidence linking ATS to leaner body composition. However, these reviews did not stratify their results or conclusions by study quality or risk of bias [12]. While previous reviews included studies that examined the relationship between ATS and cardiorespiratory fitness, the authors were unable to determine the association due to limited evidence. Since the publication of these reviews there has been an increase in the number of studies exploring this relationship.

It has been suggested that much of the activity completed by children and adolescents is of insufficient volume and intensity to improve health-related fitness (i.e., body composition, cardiorespiratory fitness, muscular fitness and flexibility) [13]. While the exact volume and intensity of activity required to improve fitness in young people is not clear [14], it is plausible to suggest that habitual ATS has the potential to improve health-related fitness (HRF) among youth. ATS provides a regular opportunity for young people to accumulate physical activity, but evidence to support or refute the health benefits of active transportation is limited due to the focus on assessing time spent in vigorous activity [8]. Furthermore, few studies have explored the effects of ATS on HRF among young people in longitudinal studies [15]. The aim of this review, therefore, was to systematically examine the potential health benefits associated with ATS among children and adolescents. The secondary aim was to explore the quality of studies that have examined the relationship between ATS and HRF and to determine whether study quality may have confounded these relationships.

## Methods

### Identification of studies

The Quality of Reporting of Meta-analyses statement (QUOROM) [16] provided the structure for this review. A systematic search of seven electronic databases (EMBASE, OVID MEDLINE, PsycINFO, PubMed, Scopus, SPORTDiscus and TRIS Online) was conducted in December 2009. Studies published from 1980 up to and including the search date were considered for inclusion. Individualized search strategies for the different databases included combinations of the following key words: (child* OR adolescent OR youth OR "young people") AND (walking OR "active travel" OR "active transport*" OR cycling OR riding OR "travel mode" OR trip) AND ("health-related fitness" OR "body composition" OR "obesity" OR "weight status" OR "physical fitness" OR fitness OR "cardiovascular fitness" OR "cardiorespiratory fitness" OR "aerobic fitness"). Only articles published or accepted for publication in refereed journals were considered for the review. Conference proceedings and abstracts, therefore, were not included. In the first stage of the literature search, titles and abstracts of identified articles were checked for relevance. In the second stage, full-text articles were retrieved and considered for inclusion. In the final stage, the reference lists of retrieved full-text articles were searched and additional articles known to the authors were assessed for possible inclusion.

### Criteria for inclusion/exclusion

In the first stage of the literature search, two of the four authors (DRL and PK) independently assessed the studies for inclusion. Studies were considered to be eligible for inclusion according to the following criteria: (i) participants were children or adolescents aged 5 to 18 years, (ii) study reported active school transportation in children and/or adolescents, (iii) study assessed at least one component of health-related fitness (i.e. cardiorespiratory fitness, muscular fitness, body composition or flexibility), (iv) study included quantitative analysis of the relationship between ATS and at least one component of health-related fitness, (v) study published in English.

### Criteria for assessment of study quality

Two authors (DRL and PK) independently assessed the quality of the studies that met the inclusion criteria (Table 1). The criteria for assessing the quality of the studies were adapted from the Strengthening the Reporting of Observational Studies in Epidemiology (STROBE) statement [17]. Items that were considered to be the most important in relation to study bias were included in the concise checklist. A formal quality score for each study was completed for a 6-item list by assigning a value of 0 (absent or inadequately described) or 1 (explicitly described and present) to each of the following questions listed: (i) Did the study describe the participant eligibility criteria? (ii) Were the study schools/participants randomly selected (or representative of the study population)? (iii) Did the study report the sources and details of ATS assessment and did the instruments have acceptable reliability for the specific age group? (iv) Did the study report the sources and details of HRF assessment and did all the methods have acceptable reliability? (v) Did the study report a power calculation and was the study adequately powered to detect hypothesized relationships? (vi) Did the study report the numbers of individuals who completed each of the different measures and did participants complete at least 80% of measures? Differences were resolved by a third reviewer (CEF).

**Table 1:** ATS study quality checklist with quality scores assigned

Studies	(i) Did the study describe the participant eligibility criteria?	(ii) Were the study schools/participants randomly selected (or representative of the study population)?	(iii) Did the study report the sources and details of ATS measurement and did the methods have acceptable reliability for the specific age group?	**(iv) Did the study report the sources and details of HRF assessment**^**a **^**and did the all of the methods have acceptable reliability for the specific age group?**	(v) Did the study report a power calculation and was the study adequately powered to detect hypothesized relationships?	**(vi) Did the study report the numbers of individuals who completed each of the different measures**^**b **^**and did participants complete at least 80% of measures?**	Quality score total/6
Evenson et al [19]	1	1	0	0	0	1	3
Tudor-Locke et al [8]	1	1	0	1	0	0	3
Metcalf et al [26]	1	0	0	1	1	0	3
De Bourdeauhuij et al [30]	0	1	1	0	0	1	3
Fulton et al [21]	0	1	0	0	0	1	2
Gordon-Larsen et al [20]	1	1	0	1	0	1	4
Heelan et al [43]	1	0	1	1	1	0	4
Klein-Platat et al [31]	1	1	1	1	0	0	4
Sirard et al [22]	1	1	0	1	0	0	3
Cooper et al [32]	1	1	0	1	0	1	4
Mota et al [35]	1	0	0	1	0	1	3
Rosenberg et al [23]	1	0	0	1	0	1	3
Timperio et al [36]	1	1	1	1	0	1	5
Ford et al [27]	1	0	0	1	0	0	2
Li et al [38]	1	1	0	1	0	0	3
Mota et al [34]	1	0	0	1	0	1	3
Ortega et al [39]	1	1	0	1	0	0	3
Saksvig et al [24]	1	1	1	1	0	0	4
Cooper et al [33]	1	1	0	1	0	1	4
Landsberg et al [40]	1	0	1	1	0	1	4
Robertson-Wilson et al [41]	1	1	1	1	0	1	5
Silva and Lopez [42]	1	1	1	1	1	1	6
Yeung et al [37]	1	0	0	0	0	1	2
Andersen et al [15]	1	1	0	1	0	0	3
Baig et al [28]	1	0	1	1	1	1	5
Madsen et al [25]	1	0	0	1	0	1	3
Voss et al [29]	0	1	0	1	0	1	3

*Note*. 1 = Yes and 0 = No; ATS = active travel to school.

^a^The measurement of height and weight using standardized procedures to calculate BMI satisfied this criteria.

^b^Studies were required to report the number of participants who completed ATS questionnaire and HRF measures separately.

### Categorization of variables and level of evidence

The level of association between ATS and HRF was determined using the coding system first described by Sallis et al. [18] (Table 2). The relationship between ATS and HRF components was determined by examining the percentage of studies that reported a statistically significant relationship (i.e. between ATS and the HRF component). If only 0-33% of the included studies reported a statistically significant relationship between ATS and a given HRF component, the result was categorized as no association (0). If 34-59% of the studies reported statistically significant relationships between ATS and the HRF component, the result was categorized as indeterminate (?). If 60-100% of studies reported a positive relationship between ATS and the HRF component, the result was coded as a positive association (+). If 60-100% of studies reported a negative relationship between ATS and the HRF component, the result was coded as a negative association (-). The methods of Sallis et al. [18] were modified to address the issue of study quality, and additional coding was conducted based on studies identified as high quality. If 60-100% of high quality studies (≥ 4) found a relationship between ATS and a given HRF component, the result was coded as having strong evidence for a positive association (++) or negative association (--).

**Table 2:** Summary of studies examining the relationship between ATS and HRF in youth

Benefits	Associated with ATS		Not associated with ATS	**Summary coding**^a^	
	
	**Reference no**.	**Assoc. (-/+)**^b^	**Reference no**.	***n*/N (%)**^c^	**Assoc. (-/+/?)**^d^
Weight status/body composition	[19,8]^e^,[20,30,43,31,23]^e^,[38,39]^f^,[40,42,25]	-	[21,26,22,35,34,24,41,37,15,28], [29]	12/25 (48%)	?
Cardiorespiratory fitness	[15]^g^,[32]^g^,[33]^g^,[29]	+	[25]	4/5 (80%)	+
Muscular fitness	[15]	+		1/1 (100%)	?
Flexibility	[15]^h^	+		1/1(100%)	?

*Note*. ATS = active travel to school; A positive or negative association was noted if at least one component of ATS was associated with the HRF component

^a^Summary code provides an overall summary of the findings for each relationship.

^b^Association shows the direction of the individual association.

^c^N = number of studies that examined and reported possible associations between ATS and HRF component, *n *= number of studies that support the relationship.

^d^Association for overall findings for each relationship for high quality studies. If 60-100% of high quality studies (≥ 4) found a relationship between ATS and HRF component, the result was coded as having strong evidence for a positive association (++) or negative association (--). If < 4 studies available the relationship was coded as indeterminate (?).

^e^Relationship for boys only.

^f^Relationship for girls only.

^g^Relationship between CRF and cycling.

^h^Cyclists had better flexibility than walkers and those who used PTS.

## Results

### Overview of studies

**Figure 1:**
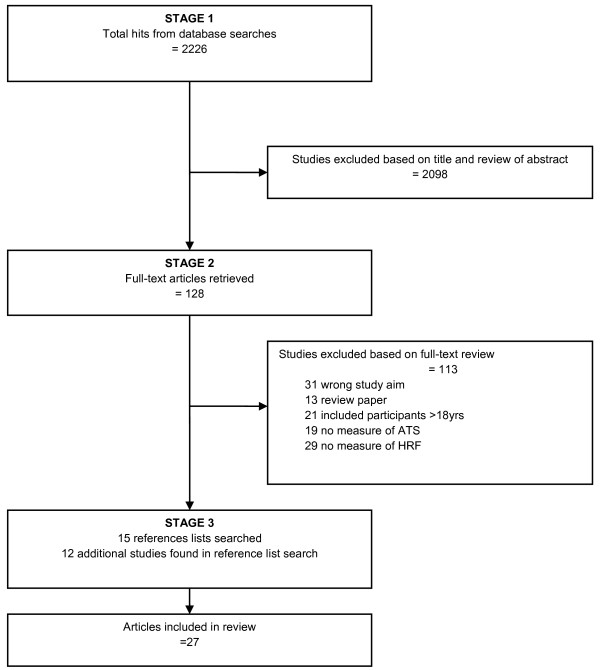
**Flow diagram of study selection**. *Note*. ATS = active travel to school, HRF = health-related fitness

The flow of studies through the search process is reported in Figure 1. While the majority of studies (Table 3) were conducted in the US [19-25], the relationship between ATS and HRF was explored in a further 12 different countries, including the Philippines [8], England [26-29], Holland [30], France [31], Denmark [15,32,33], Portugal [34,35], Australia [36,37], China [38], Spain [39], Germany [40], Canada [41], and Brazil [42]. The study sample sizes ranged from 107 [37] to 21,345 [41]. While most of the studies involved cross-sectional study designs, Cooper and colleagues [33] examined longitudinal changes in transport to school over a 6-year study period. Similarly, Rosenberg et al [23] explored the relationship between school travel and weight status over a 2-year period. The prevalence of ATS among study participants ranged from 3 and 8% in boys and girls [22], to 72 and 79% in boys and girls [15].

**Table 3:** Summary of included studies

Study	Sample	Study design	ATS classification	HRF component(s) and method of assessment	Analyses	Results	Prevalence of ATS
Evenson et al [19]	4448 adolescents Grades 6-12 United States	Cross-sectional	Walking or riding to/from school at least once/week	BC- Self report BMI z-score (>85^th ^percentile considered overweight)	Logistic regression	Middle school students above the 85^th ^percentile were less likely to use ATS BMI categories not associated with ATS in high school students.	6^th ^grade- 12.3% 7^th ^grade- 7.5% 8^th ^grade- 8.2% 9^th ^grade- 6.0% 10^th ^grade- 5.7% 11^th ^grade- 4.0% 12^th ^grade- 3.0%
Tudor-Locke et al [8]	1518 adolescents 14-16 years Philippines	Cross-sectional	Usual travel to/from school (ATS, combination of ATS and PTS or PTS only)	BC- BMI	ANOVA	BMI not associated with ATS in girls. Boys using ATS only had significantly lower BMI values than boys using PTS only.	Boys- 47% (323/691) Girls- 37% (303/827)
Metcalf et al [26]	275 children 5 years England	Cross-sectional	Usual travel to/from school (ATS or PTS)	BC- BMI and sum of 5 skinfolds	ANOVA	No relationship between ATS and BMI or skinfolds.	Boys- 63% (97/154) Girls- 73% (88/121)
De Bourdeauhuij et al [30]	6078 children and adolescents 11-17 years Belgium	Cross-sectional	Usual travel to/from school (ATS or PTS)	BC- Self-report BMI z-score (>85^th ^percentile considered overweight)	Independent samples t-test	Overweight youth less likely to use ATS.	Not available
Fulton et al [21]	1395 children and adolescents Grades 4 to 6, 7 to 9 and 10 to 12 United States	Cross-sectional	Usual travel to/from school or work (ATS or PTS)	BC- Proxy and self-report BMI	Logistic regression	No association between BMI and ATS.	Boys- 17% (121/727) Girls- 11% (74/668)
Gordon-Larsen et al [20]	10771 adolescents Grades 7 to 12 United States	Cross-sectional	Usual travel to/from school or work (ATS or PTS)	BC- BMI	Independent samples t-test	Rates of ATS were higher among non-overweight adolescents.	
Heelan et al [43]	320 children 10.2 years United States	Cross-sectional	How they travelled to/from school in the past 24 hours (ATS or PTS) and amount of time taken	BC- BMI, average of 3 skinfolds	Multiple regression	Significant association between BMI and ATS in overweight children. No association between skinfolds and ATS.	Boys & girls- 33.3% (107/320)
Klein-Platat et al [31]	2714 adolescents 12 years France	Cross-sectional	Walking or riding to/from school- none, 0-20 min/day and > 20 min/day	BC- BMI z-score (>90^th ^percentile considered overweight) and WC	ANCOVA	ATS associated with weight status and WC.	Boys- 40% (543/1357) Girls- 37% (503/1357)
Sirard et al [22]	219 children 10.3 years United States	Cross-sectional	Walking or riding to/from school- regular active commuters (>5 times/week), irregular active commuters (1-4 times/week) or passive commuters (0 times/week)	BC- BMI z-score (>85^th ^percentile considered overweight)	ANOVA	No association between weight status and ATS.	Boys- 3% (3/96) Girls- 8% (8/123)
Cooper et al [32]	529 children and 390 adolescents 9.7 and 15.5 years Denmark	Cross-sectional	Usual travel to/from school [passive (car, motorcycle, train, bus), bicycle or walk] and duration of journey	CRF- Progressive cycle ergometer	ANOVA	Children and adolescents who cycled to school had higher CRF than those who used PTS. No relationship between walking to school and CRF.	Boys (child)-66% (168/254) Girls (child)- 62% (172/276) Boys (adol)-87.5% (169/193) Girls (adol)-85% (167/196)
Mota et al [35]	450 adolescents 14.6 years Portugal	Cross-sectional	Usual travel to/from school (ATS or PTS) and duration of journey	BC- BMI z-score (>85^th ^percentile considered overweight)	Bivariate correlation	No association between weight status and ATS.	Boys & girls- 23.1% (105/450)
Rosenberg et al [23]	1083 children Grades 4 and 5 United States	Longitudinal	ATS- 2 or more days/week of ATS at each of the 4 assessment periods.	BC- BMI z-score (>85^th ^percentile considered overweight), average of 3 skinfolds	ANOVA	Boys who used ATS at baseline had significantly lower BMI and skinfolds than passive commuters. No association between ATS and weight status in girls. No association between changes in BMI and skinfolds and ATS over 2-year period.	Boys- 36% (116/320) Girls- 29% (79/274)
Timperio et al [36]	912 children 5 to 6 and 10 to 12 years Australia	Cross-sectional	ATS at least once per week	BC- BMI z-scores (IOTF classification)	Logistic regression	No association between ATS and weight status.	Boys- 9% (29/316) Girls- 4% (13/361)
Ford et al [27]	239 children 5 to 11 years England	Cross-sectional	PST or ATS (walk) for > 10 mins, more than 3 times/week for at least 15 weeks	BC- Air displacement plethysmorgaphy (BodPed Self-Test)	Mann-Whitney U test	No significant difference in body fat between ATS a	Boys- 47% (59/125) Girls- 43% (49/114)
Li et al [38]	6826 children and adolescents 7 to 17 years China	Cross-sectional	Usual travel to/from school- walking or riding classified as ATS	BC- BMI z-score (>85^th ^percentile considered overweight)	Cox regression analysis controlling for parental overweight and SES	Overweight youth less likely to use ATS.	Boys & girls- 93.6% (6386/6826)
Mota et al [34]	705 adolescent girls 14.7 years Portugal	Cross-sectional	Usual travel to/from school- walking or riding classified as ATS	BC- BMI	Chi-square and bivariate correlation	No association between BMI and ATS.	Boys & girls- 52.6% (371/705)
Ortega et al [39]	2859 adolescents 13 to 18.5 years Spain	Cross-sectional	ATS classified as riding/walking to/from school > 15 min/day	BC- BMI z-scores, WC adjusted for height	ANCOVA	Weights status not associated with ATS in boys or girls. Significant association between ATS and WC in girls.	Boys- 9.6% (130/1357) Girls- 13% (189/1502)
Saksvig et al [24]	1721 adolescent girls 12.0 years United States	Cross-sectional	Travel by walking on 1 or more weekdays before/after school.	BC- BMI	Linear mixed models	No difference in BMI among those who walked to school and those who did not.	Before school - 13.6% (232/1701) After school 17.7% (301/1701)
Cooper et al [33]	384 children 9.7 years Denmark	Longitudinal	Usual travel to/from school (cycle, walk or PTS).	CRF- Progressive cycle ergometer	ANOVA	CRF was significantly higher among children and adolescents who cycled to school at one or both time periods compared to those who used other forms of transport.	Boys- 66% (110/170) Girls- 65% (137/214)
Landsberg et al [40]	626 adolescents 14 years Germany	Cross-sectional	Usual travel to/from school (ATS or PTS) and duration of journey.	BC- BMI z-score (>85^th ^percentile considered overweight), Sum of 4 skinfolds (triceps, biceps, suprailiacal and subscapular), bioelectrical impedance (fat mass) and WC.	General linear models	ATS associated with lower fat mass and skinfolds. ATS associated with BMI or WC.	Boys- 63% (206/328) Girls- 50% (163/298)
Robertson-Wilson et al [41]	21345 adolescents Grades 9 to 12 Canada	Cross-sectional	Usual travel to/from school (ATS or PTS).	BC- BMI z-score (>85^th ^percentile considered overweight)	Binary logistic regression	No association between ATS and weight status.	Boys- 44% (4699/10747) Girls- 41% (4378/10598)
Silva and Lopez [42]	1570 children 7 to 12 years Brazil	Cross-sectional	Usual travel to/from school (ATS or PTS) and duration of journey.	BC- BMI z-scores (IOTF classification) and skinfolds (tri-cipital)	Regression	ATS was associated with a lower prevalence of excess weight and body fat.	Boys- 15% (117/785) Girls- 18% (131/742)
Yeung et al [37]	107 children 4 to 12 years Australia	Cross-sectional	ATS at least once/week.	BC- Parental proxy BMI	Mann-Whitney U test	No association between BMI and mode of transportation to school.	Boys- 40% (59/149) Girls- 27% (46/169)
Andersen et al [15]	1249 adolescents 15 to 19 years Denmark	Cross-sectional	Usual travel to/from school (cycle, walk or PTS).	BC- BMI CRF- Progressive cycle ergometer MF- sit-ups, static back strength, arm flexion dynamic test FL- sit and reach	ANOVA	Cyclists had higher CRF, MF and FL than both walkers and those who use PTS. No relationship between BMI and transportation mode.	Boys- 72% (391/545) Girls- 79% (559/704)
Baig et al [28]	673 adolescents 12.6 years England	Cross-sectional	Usual travel to/from school (cycle, walk, public transport or bus) and duration of journey calculated.	BC- BMI z-scores (IOTF classification)	Binary logistic regression	No association between ATS and weight status.	Girls 3.6 times more likely to walk to school than boys
Madsen et al [25]	5357 adolescents Grades 7 and 9 United States	Cross-sectional	Travel to (day of survey) and from (day before survey) school.	BC- BMI z-scores (CDC >85^th ^percentile considered overweight) CRF- 1 mile run test	Linear regression	ATS was inversely associated with weight status A non-significant (*p *= 0.07) trend between ATS and CRF was found.	To school - 29% (1554/5357) From school - 46% (2464/5357)
Voss et al [29]	6085 children and adolescents 10 to 15.9 years England	Cross-sectional	Usual travel to/from school (cycle, walk, public transport or bus) and duration of journey calculated.	BC- BMI z-scores (IOTF classification) CRF- 20 m shuttle run test	ANOVA	No association between travel mode and BMI. ATS associated with improved CRF.	Boys- 59% (1845/3135) Girls- 57% (1587/2792)

ATS = Active travel to school; BC = Body composition; BMI = Body mass index; CDC = Centers for Disease Control & Prevention; CRF = Cardiorespiratory fitness; FL = Flexibility HRF = Health-related fitness; IOTF = International Obesity Task Force; MF = Muscular fitness; NR = Not reported; PTS = Passive travel to school; WC = Waist circumference.

### Overview of study quality

There was 90% agreement between reviewers (DRL and PK) on the study assessment criteria and full consensus was achieved with the help of a third reviewer (CEF). Eleven studies scored ≥ 4 on the study quality assessment [20,24,28,31-33,36,40-43] (Table 1). The majority of studies used samples of schools/participants who were randomly selected and/or representative of the study population. Most of the studies described the participant eligibility criteria, although participant eligibility (e.g. were participants selected from all grades at participating schools?) was unclear in three studies [21,29,30]. The majority of studies reported standardized procedures for the assessment of height, weight and cardiorespiratory fitness. Two studies reported parental proxy reports for their children's height and weight and did not provide reliability/validity data [21,37] and a further two studies used self-reported height and weight to calculate BMI [19,30]. While the majority of studies involved large sample sizes and were adequately powered to detect hypothesized relationships, only two studies reported power calculations relevant to their study aims [42,43]. Fifteen studies reported the number of individuals who completed each of the different measures.

### Overview of findings

Twenty three studies examined the relationship between ATS and weight status/body composition (Table 2). ATS was associated with more beneficial weight status (or lower body fat) in 11 (48%) of these studies. However, when lower quality studies (< 4) were excluded from the summary, five of the nine remaining studies found ATS to be beneficially associated with weight status. Five studies examined the association between ATS and CRF [15,25,29,32,33]. Four of these studies [15,29,32,33] found a positive association, while the fifth study [25] found a non-significant (*p *< .07) trend between ATS and CRF. More specifically, three of four studies involving cycling reported positive associations with CRF, and a 6-year longitudinal study [33] demonstrated that change in travel mode to school from non-cycling to cycling was a significant predictor of CRF at follow-up. Further evidence supporting the benefits of cycling was provided by Andersen and colleagues [15], who found that muscular fitness and flexibility were significantly better among Danish cyclists compared to walkers and those who used passive transportation.

## Discussion

The aim of this review was to systematically examine the potential health benefits associated with ATS among children and adolescents. Of the 27 studies that were included in the review, 25 examined the relationship between ATS and weight status/body composition. While only 48% of these studies reported significant inverse associations between ATS and weight status/body fatness, this increased to 55% once poor quality studies were removed. Based on the findings from five studies, including one longitudinal study, it appears that ATS may be associated with superior cardiorespiratory fitness in youth. It was not possible to draw any conclusions about the associations between ATS, muscular fitness and flexibility due to the small number of studies exploring these relationships.

Despite methodological differences, these results are similar to those of previous reviews [9,10]. Two previous ATS and health reviews reached the same conclusion about a lack of association between body weight and ATS despite not assessing the quality of their included studies. The decision to use an assessment of study quality or risk of bias was both pragmatic and justified. The application of this metric allowed for the removal the studies with the greatest risk of bias. Faulkner and colleagues' review [10] reported just over half the studies included in this review (n = 10), that had examined associations between ATS and body weight. One explanation for this difference lies in the recent increase in ATS-related publication since their review's search date (06/2008). Their review not only focused on the relationship between ATS and overall physical activity, but also adopted inclusion criteria for studies for the assessment of physical activity using an objective measure (pedometer or accelerometer). Despite these differences, the findings of both reviews were consistent, concluding that there is little evidence to suggest an association between body weight and ATS.

Previous reviews that examined the relationship between ATS and cardiorespiratory fitness were unable to draw conclusions due to the small number of studies. However, evidence from a number of recent well designed studies suggests a positive association between ATS and cardiorespiratory fitness. One partial explanation for these positive associations may be the use of cycle ergometers to measure fitness in three of the five studies, which may have favored those children who cycled to school [15,32,33]. However, the two other studies that used running-based fitness assessments also observed differences in fitness between ATS and non ATS groups, with one significant [29] and one borderline nonsignificant [25] result. Furthermore, a recent study, not included in the current review, found that CRF levels were higher among young Swedish and Estonians adolescents who cycled to school compared to those who walked or used passive transportation methods [44]. There appears, therefore, to be some evidence supporting the observation that fitness is associated with ATS, but future studies must account for loss to follow-up and validated assessments of ATS to improve confidence in these observations.

Similar limitations to the overall quality of studies were observed, particularly in the definition and measurement of ATS. There were a range of definitions for classifying a participant as an active traveler. These definitions used different categories for frequency, duration and type of activity that counted as ATS. For example, studies often used questions about "usual mode of travel to and from school", and only 11 of the 27 studies reported an acceptable reliability of their methodology for ATS assessment in the sample population. Thus, standardized definition and measurement should be addressed in future studies, as active travel is a collection of behaviors that vary by purpose and duration [45], e.g. journey to and from school. The potential for misclassification of ATS was also reflected in the actual measurement of ATS mode, with no study reporting a validated measure of ATS by travel mode. With the advent of both portable global positioning system devices and digital image capture systems, the opportunity to improve this potential source of bias must be addressed. These new systems, which allow the confirmation of journey mode, duration and distance, are currently being evaluated in a number of international studies [46,47]. Such systems would also address the issue of possible confounding or mediation of ATS behavior and health outcome by the built environment characteristics of study areas [48].

The majority of studies did not examine associations by population sub- groups, e.g. gender or age. Indeed, study populations were treated as homogeneous, being usually dichotomized into ATS v non-ATS groups. Such an approach is limited as it ignores the broader social, environmental and personal level correlates of behavior. Indeed, Panter and colleagues [48] highlighted the importance of facilities to assist active travel and urban design in the neighborhood, as well as shorter distances and road safety in-route in relation to active travel. A recent review of qualitative studies of children's experiences of active travel not only supported these observations, but also highlighted the importance of the constraining influence of parents' restrictions on independent movement, as well as children's own fears of traffic [49]. All these factors clearly have a major impact on children's ability to undertake ATS and may provide an explanation why some studies fail to report consistent associations [9,10]. Only two studies reported power calculations in their methods sections. While the majority of the studies included large samples and were likely to have adequate power to detect hypothesized relationships, the reporting of power calculations should be considered by others in the future.

Body composition/weight status was measured in a variety of ways, with most studies employing BMI z-scores or percentiles to classify youth as healthy, overweight or obese. Two studies [21,37] used parental proxy reports of their children's height and weight and both studies found no association between BMI and method of transportation to school. Alternatively, two studies [19,30] used students' self-reported BMI and found significant associations between ATS and weight status. More consistency in using a more objective assessment of this exposure variable in future studies would help to establish the association in relation to ATS.

This is the first systematic review to report the relationship between ATS and HRF in youth. In addition, the assessment of study eligibility and quality was conducted independently by two authors and consensus was reached for every decision. However, the limitations of this review should be noted. First, the measurement and classification of ATS were different across studies, which may explain some of the inconsistencies found. Second, the quality assessment was not extensive and additional criteria may provide additional insights. Finally, the search strategy was limited to published studies identified through the selected search engines. As more studies continue to be published, it will be important to reconsider and refine these findings. Despite these shortcomings, it seems that the majority of higher quality studies reported consistent associations between ATS and the important health indexes of weight status/body composition and cardiorespiratory fitness. With such health benefits for children, the promotion of ATS provides a real opportunity for public health interventions to tackle the double challenge of obesity and poor fitness.

## Conclusions

There is some evidence to suggest that active travel to school is associated with a healthier body composition and level of cardiorespiratory fitness among youth. However, as this review included only two longitudinal studies, more interventions and longitudinal studies exploring the relationship between changes in health-related fitness and active travel are needed to investigate the causal nature of such relationships. While it is important that researchers continue to monitor children and adolescents school transportation behaviours, there is a need to broaden the research on young people's active transport to include journeys to destinations other than school, e.g. walking/cycling trips from home to a variety of neighbourhood destinations. Distance to school has been identified as a barrier to active transportation [50] and public health strategies promoting active transportation to more proximal destinations are clearly warranted.

## Competing interests

The authors declare that they have no competing interests.

## Authors' contributions

DRL and CEF conducted the literature search. DRL and PK evaluated the quality of included studies. All authors contributed the editing and approving of the final version of the paper.
